# Supplementary material for: Multisensory correlation computations in the human brain identified by a time-resolved encoding model
Source: Nat Commun. 2022 May 5;13:2489. doi: 10.1038/s41467-022-29687-6 (PMC9072402; doi:10.1038/s41467-022-29687-6)
Supplement: Supplementary file 1 — Supplementary Information [file 41467_2022_29687_MOESM1_ESM.pdf]

Multisensory correlation computations in the human brain identified by a time-resolved encoding model

Jacques Pesnot Lerousseau[1,3,4,*], Cesare Parise[2], Marc O. Ernst[3] and Virginie van Wassenhove[4]

[1] Aix Marseille Univ, Inserm, INS, Inst Neurosci Syst, Marseille, France
[2] Independent researcher
[3] Applied Cognitive Psychology, Ulm University, Ulm, Germany
[4] Cognitive Neuroimaging Unit, CEA DRF/Joliot, INSERM, CNRS, Université Paris-Saclay, NeuroSpin, 91191 Gif/Yvette, France.
* Correspondence: jacques.pesnot-lerousseau@univ-amu.fr

[SUPPLEMENTARY FIGURES AND TABLES]

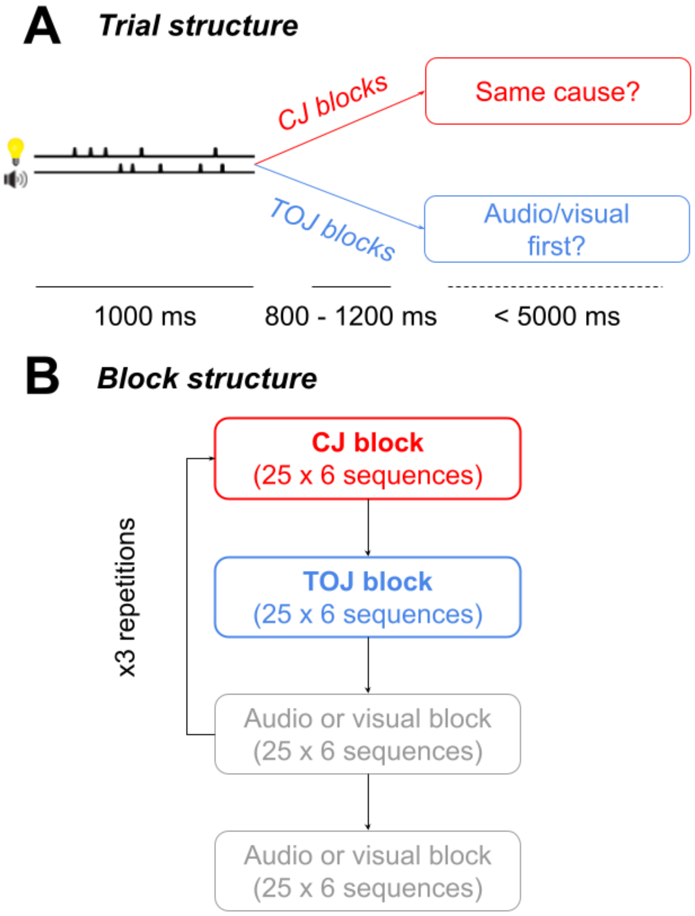

Supplementary Figure 1. Experimental Paradigm. A. We presented participants with sequences of 5 auditory clicks and 5 visual flashes of varying temporal structure. Each trial was composed of a 1000 ms sequence followed by a period of silence of varying duration (800 - 1200 ms). Participants were then prompted to respond via a probe sound (1 kHz pure tone, 10 ms). In the causality judgment task, participants judged whether the presented audiovisual sequences originated from the same source. In different experimental blocks, participants reported which of the acoustic or visual events were first in the sequence. No feedback was provided. **B.** The experiment was composed of 10 experimental blocks. During the 3 causality and 3 temporal order judgment blocks, participants had to do the task and use button presses to respond. During the two AUDIO and VISUAL localizer blocks, participants were asked to pay attention to the stimuli, without responding.

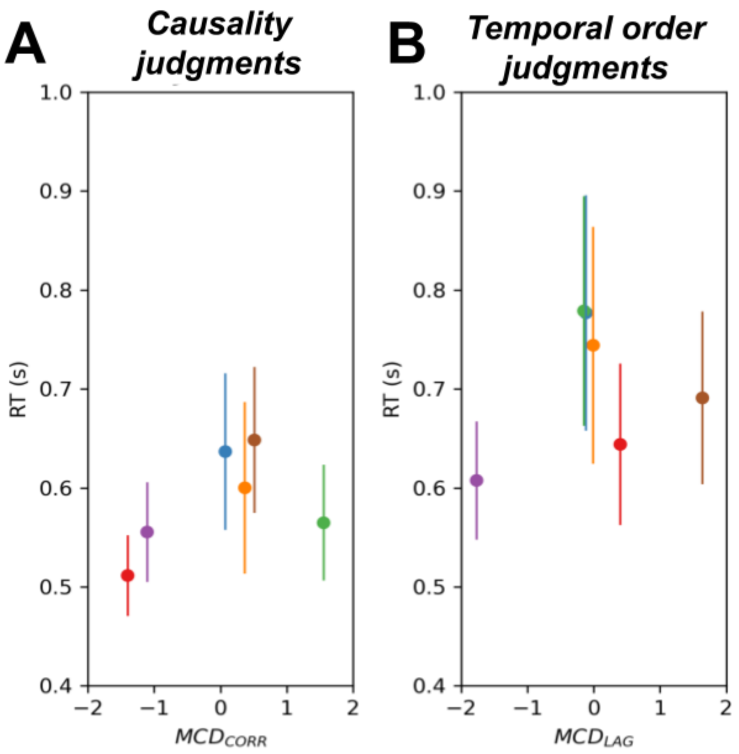

Supplementary Figure 2. Unexpected differences in reaction times in causality and temporal order judgment blocks. In a given trial, following the presentation of an audiovisual sequence, participants were asked to withhold their answers until an auditory cue prompted them to answer. The Multisensory Correlation Detector model makes no explicit predictions about reaction times. However, we observe that, although stimuli are strictly identical, reaction times differ as a function of the task. Nonetheless, considering that the response delay was 1s, it is likely not long enough to completely dissociate action selection from sensory processes. Reaction times in the **A.** Causality judgment blocks were systematically lower than reaction times in the **B.** Temporal order judgment blocks (mixed-model linear regression, $p < 10^{-15}$). Error bars represent 2 s.e.m. across participants (N=13).

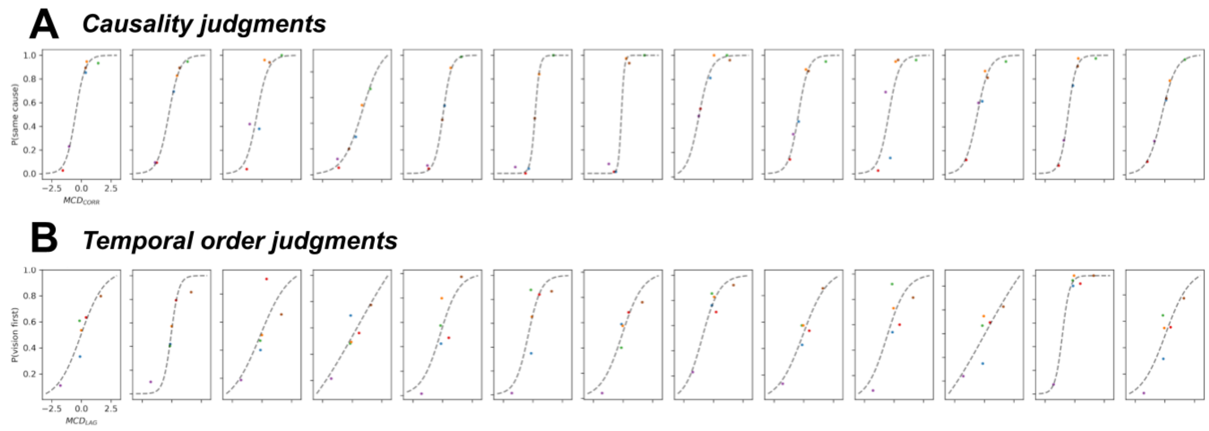

Supplementary Figure 3. Individual behavioral data and fits by the MCD model. A. Individual fits for the causality judgments by the MCD_{CORR}. **B.** Individual fits for the temporal order judgments by the MCD_{LAG}.

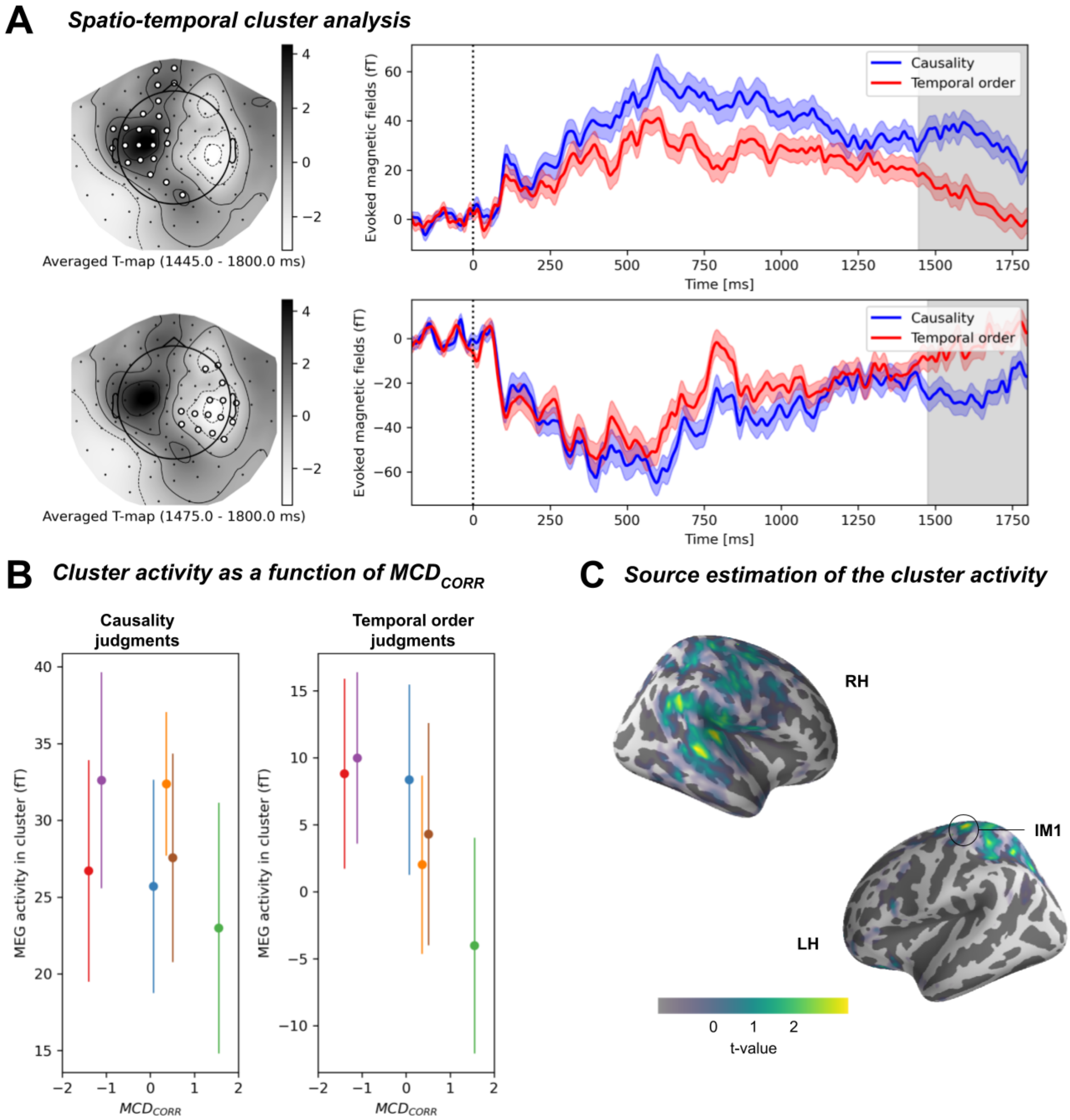

Supplementary Figure 4. Late task related evoked activity and cortical source estimates. A. Spatiotemporal-cluster analysis contrasting brain activity evoked by the presentation of identical audiovisual sequences but in two different tasks (causality - temporal order). In the contrast of causality *vs*. temporal order judgment blocks, a second bilateral cluster was found from central to frontal sensors (white sensors, one sample two-sided t-test, $p < 0.05$ corrected for multiple comparison). The peak significance was around 1600 ms, and lasted from 1445 ms to the end of the considered epochs ([1445 - 1800 ms] for the left hemisphere; [1475 - 1800 ms] for the right hemisphere). During this time window, the amplitude of the signal was consistently higher in the causality judgment blocks than in the temporal order judgment blocks. The top and bottom panels represent the two polarities of a single source (positive left, negative right). **B.** A logistic regression on the probability of a response showed no link between activity in the cluster and behavior (all $p > 0.9$). **C.** Source reconstruction revealed that this cluster was located in the left motor and left premotor cortices as well as in bilateral Superior Parietal Gyrus (SPG). This was consistent with the side of participants' right hand response and the response prompt occurring

between 1800 and 2200 ms. Due to the prompting of participants to respond and the task instructions focusing on accuracy, we did not expect differences in RTs between experimental conditions. However, we did find significantly faster responses in causality judgments than in temporal order judgments. This cluster indeed captures this difference ($p < 0.05$). Shaded areas and error bars represent 2 s.e.m. across participants.

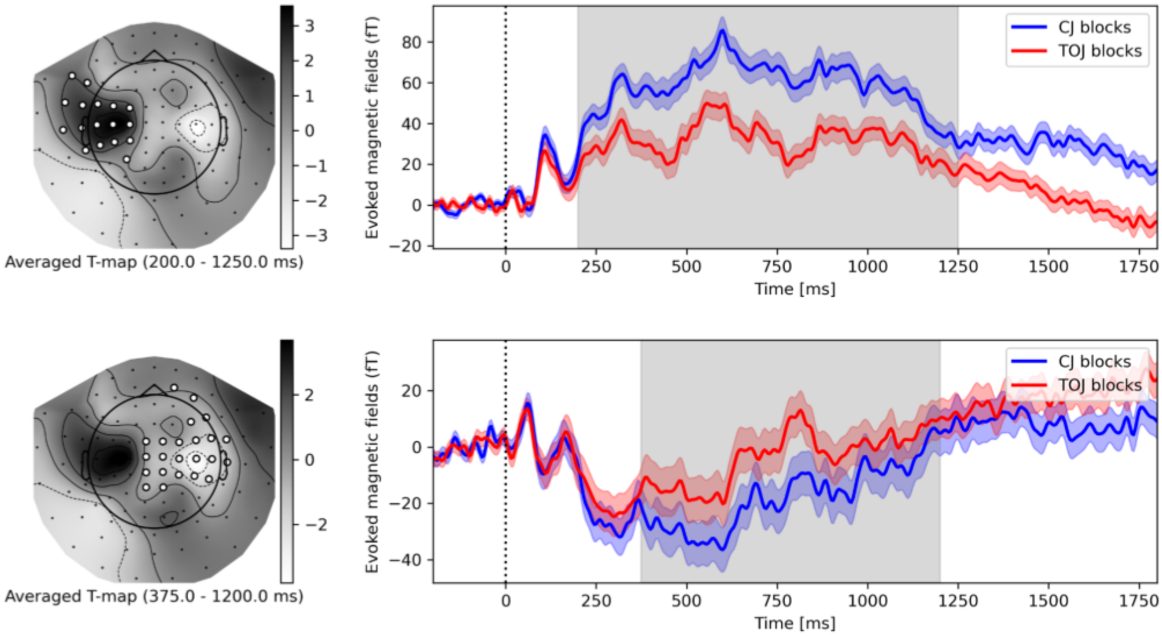

Supplementary Figure 5. Control spatiotemporal-cluster analysis using the signals without performing the removal of the unisensory-specific activity. Spatiotemporal-cluster analysis contrasting brain activity evoked by the presentation of identical audiovisual sequences but in two different tasks (causality *vs.* temporal order judgment). Left: t-map of the significant cluster (white sensors, one sample two-sided t-test, $p < 0.05$ corrected for multiple comparison) ranging from 200 to 1250 ms post-sequence onset. Grey levels are t-values averaged across significant times. Right: Temporal extent of the effect averaged over significant sensors in the cluster (grey). The top and bottom panels represent the two polarities of a single source (positive left, negative right). This analysis replicates the results presented in Fig. 3A, using the signals without performing the removal of the unisensory-specific activity. Shaded areas represent 2 s.e.m. across participants.

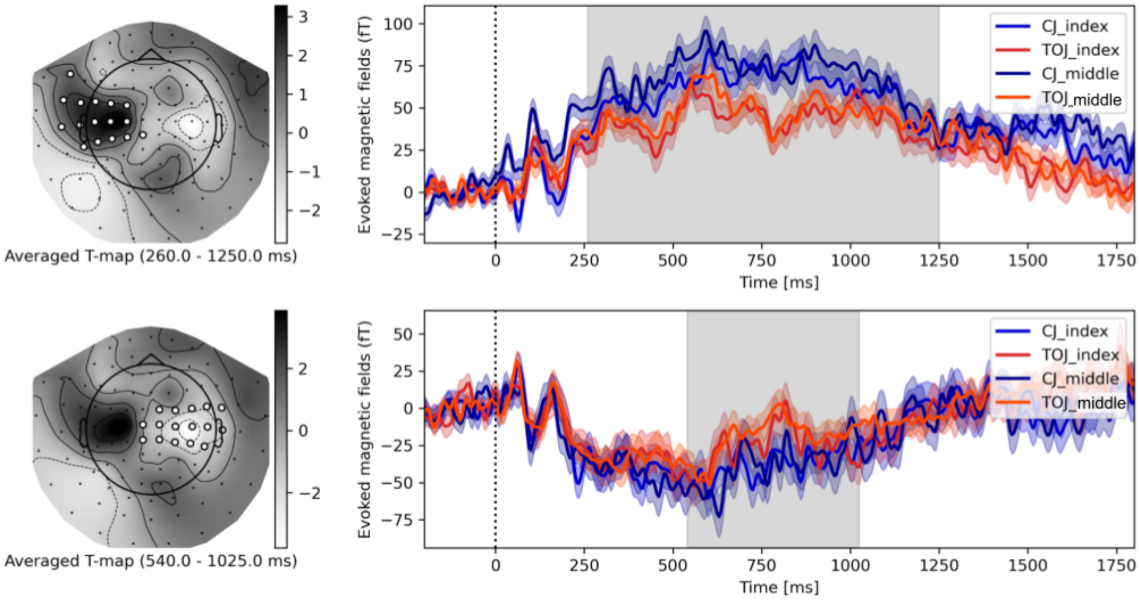

Supplementary Figure 6. Control spatiotemporal-cluster analysis splitted by fingers. Spatiotemporal-cluster analysis contrasting brain activity evoked by the presentation of identical audiovisual sequences but in two different tasks (causality *vs.* temporal order judgment). Left: t-map of the significant cluster (white sensors, $p < 0.05$ corrected for multiple comparison) ranging from 260 to 1250 ms post-sequence onset. Grey levels are t-values averaged across significant times. Right: Temporal extent of the effect averaged over significant sensors in the cluster (grey). The top and bottom panels represent the two polarities of a single source (positive left, negative right). This analysis replicates the results presented in Fig. 3A. A spatiotemporal clustering analysis on a 2 by 2 ANOVA with factor Task identity (2) and Finger identity (2) showed no significant effects of Finger identity ($p > 0.05$ for all clusters on the F-values associated with Finger identity) or interaction between Task and Finger identity ($p > 0.05$ for all clusters on the F-values associated with the interaction Task identity x Finger identity). Shaded areas represent 2 s.e.m. across participants.

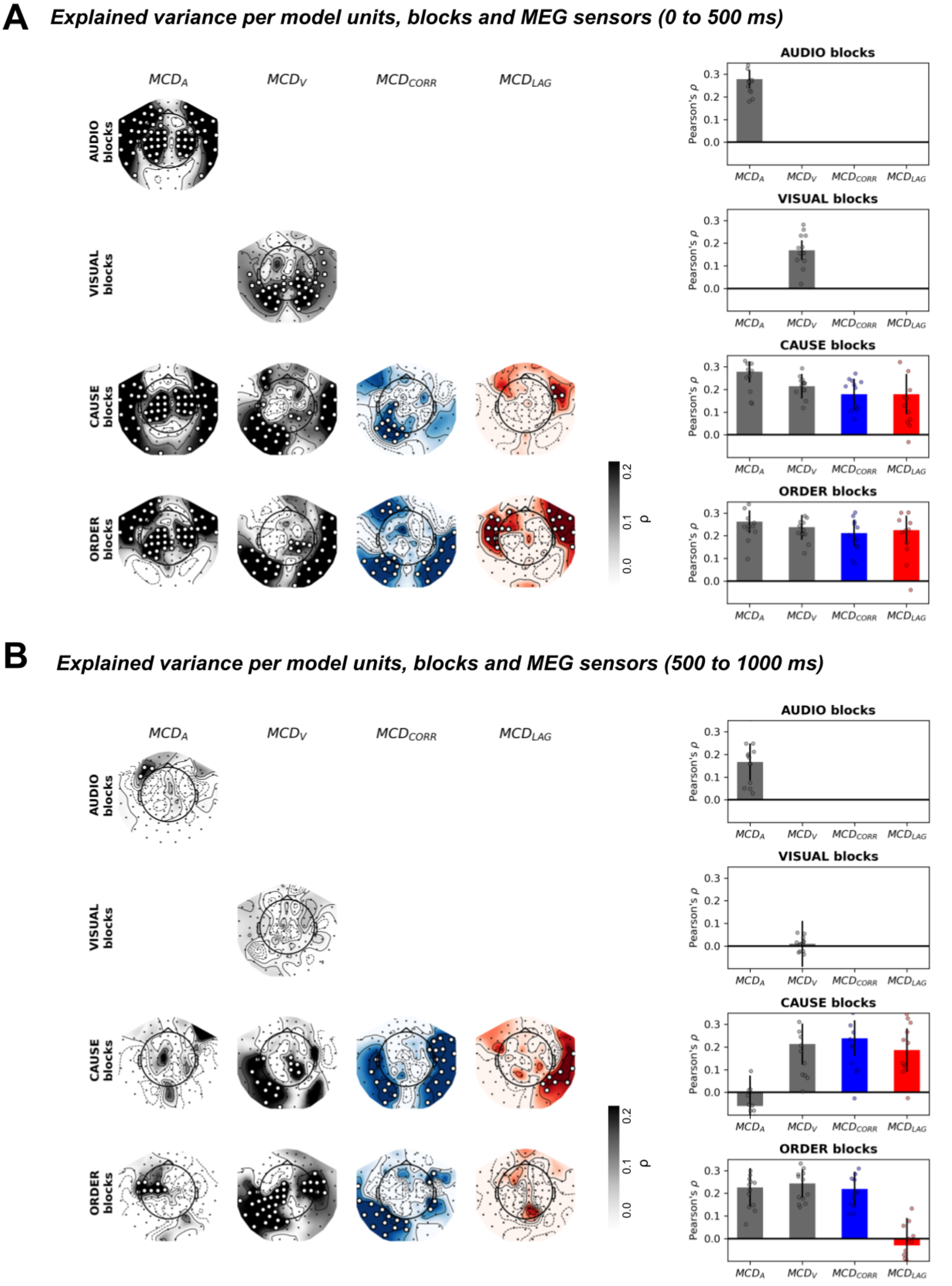

Supplementary Figure 7. Model-based temporal response functions, split analysis. A. Replication of the data presented in Fig 4 for the first half of the evoked response (from 0 to 500 ms). **B.** Replication of the data presented in Fig 4 for the second half of the evoked response (from 500 to 1000 ms). Statistical significance of the correlation between

cross-validated predicted MEG and true MEG was assessed via corrected cluster permutations. Significant sensors are highlighted in white. Error bars represent 2 s.e.m. across participants (N=13). Dots represent individual participants.

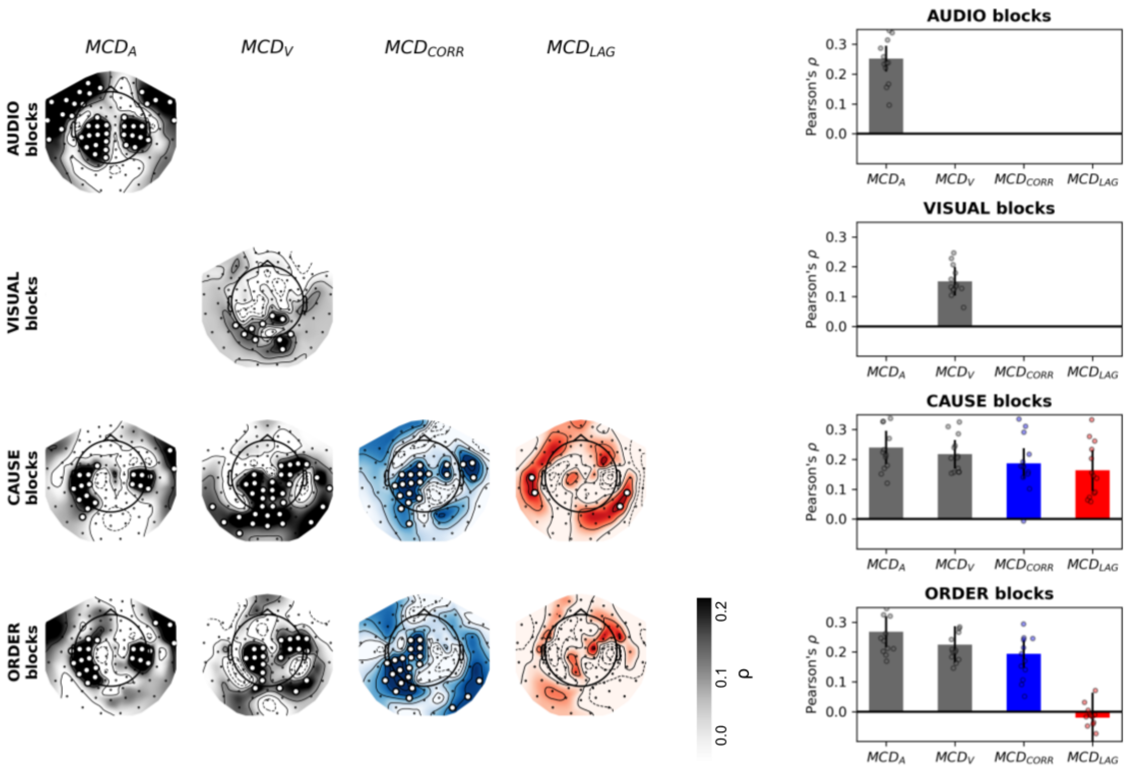

Supplementary Figure 8. Model-based temporal response functions, reduced dataset. Replication of the data presented in Fig 4 for the 4 stimuli that receive uncertain judgment in the temporal order judgment task (stimuli blue, green, orange and red in Fig. 2). Statistical significance of the correlation between cross-validated predicted MEG and true MEG was assessed via corrected cluster permutations. Significant sensors are highlighted in white. Error bars represent 2 s.e.m. across participants (N=13). Dots represent individual participants.

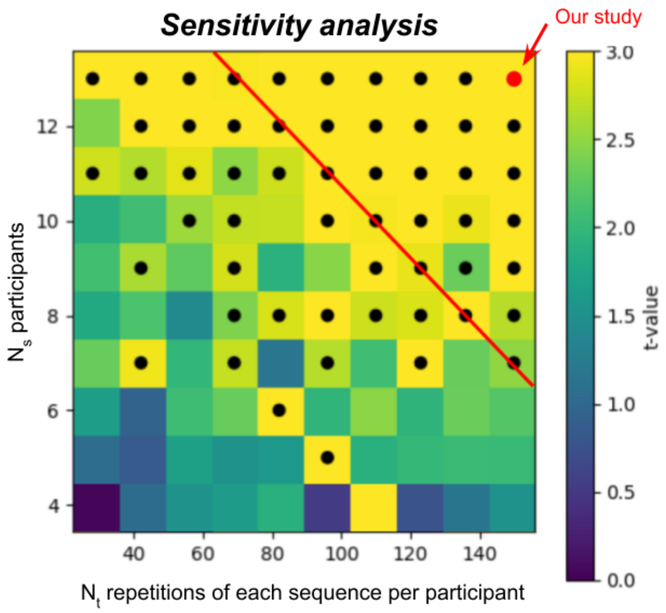

Supplementary Figure 9. Sensitivity analysis. Average t-value inside the spatiotemporal cluster for randomly selected N_s = 4, 5, …, 13 participants and N_t = 30, 45, …, 150 repetitions of each sequence. The procedure was repeated 100 times and averaged across the 100 repetitions. Any combination of number of participants and number of repetitions per participant above the red line (which corresponds to a total of 10,500 trials) would be sufficient to show an effect (we have 19,500 trials). The red dot indicates the current study. Dots (black and red) indicate a significant t-value (one sample two-sided t-test, $p < 0.05$, corrected for multiple comparisons).

Sequence #	1st sound/flash (ms)	2nd	3rd	4th	5th
1	53	194	372	511	800
	288	494	601	731	834
2	118	214	693	752	807
	163	364	524	614	940
3	338	397	534	734	844
	104	188	262	442	807
4	65	184	238	646	883
	117	184	322	413	469
5	108	298	489	739	900
	128	278	505	783	879
6	64	165	491	587	645
	58	130	363	624	788

Supplementary Table 1. Precise timing (ms) of the sequences used in the experiment.

Sequence #	MCD_{CORR}	MCD_{LAG}
**1**	15.9	-0.41
**2**	15.0	0.37
**3**	15.4	0.96
**4**	14.7	0.42
**5**	15.4	0.20
**6**	16.2	0.36

Supplementary Table 2. Unnormalized values of MCD_{CORR} and MCD_{LAG} of the sequences used in the experiment.
