# Supplementary material for: Multisensory correlation computations in the human brain identified by a time-resolved encoding model
Source: Nat Commun. 2022 May 5;13:2489. doi: 10.1038/s41467-022-29687-6 (PMC9072402; doi:10.1038/s41467-022-29687-6)
Supplement: Supplementary file 3 — Reporting Summary [file 41467_2022_29687_MOESM3_ESM.pdf]

nature portfolio

Corresponding author(s): Jacques Pesnot Lerousseau

Last updated by author(s): Feb 19, 2022

Reporting Summary

Nature Portfolio wishes to improve the reproducibility of the work that we publish. This form provides structure for consistency and transparency in reporting. For further information on Nature Portfolio policies, see our Editorial Policies and the Editorial Policy Checklist.

Statistics

For all statistical analyses, confirm that the following items are present in the figure legend, table legend, main text, or Methods section.

n/a	Confirmed	
☐	☒	The exact sample size (*n*) for each experimental group/condition, given as a discrete number and unit of measurement
☐	☒	A statement on whether measurements were taken from distinct samples or whether the same sample was measured repeatedly
☐	☒	The statistical test(s) used AND whether they are one- or two-sided<br>*Only common tests should be described solely by name; describe more complex techniques in the Methods section.*
☐	☒	A description of all covariates tested
☐	☒	A description of any assumptions or corrections, such as tests of normality and adjustment for multiple comparisons
☐	☒	A full description of the statistical parameters including central tendency (e.g. means) or other basic estimates (e.g. regression coefficient) AND variation (e.g. standard deviation) or associated estimates of uncertainty (e.g. confidence intervals)
☐	☒	For null hypothesis testing, the test statistic (e.g. F, t, r) with confidence intervals, effect sizes, degrees of freedom and P value noted<br>*Give P values as exact values whenever suitable.*
☒	☐	For Bayesian analysis, information on the choice of priors and Markov chain Monte Carlo settings
☐	☒	For hierarchical and complex designs, identification of the appropriate level for tests and full reporting of outcomes
☐	☒	Estimates of effect sizes (e.g. Cohen's d, Pearson's r), indicating how they were calculated

Our web collection on statistics for biologists contains articles on many of the points above.

Software and code

Policy information about availability of computer code

Data collection	Matlab (R2012a, Mathworks Inc.)
Data analysis	Python 3.5<br>MNE_python 0.24

For manuscripts utilizing custom algorithms or software that are central to the research but not yet described in published literature, software must be made available to editors and reviewers. We strongly encourage code deposition in a community repository (e.g. GitHub). See the Nature Portfolio guidelines for submitting code & software for further information.

Data

Policy information about availability of data

All manuscripts must include a data availability statement. This statement should provide the following information, where applicable:

- Accession codes, unique identifiers, or web links for publicly available datasets
- A description of any restrictions on data availability
- For clinical datasets or third party data, please ensure that the statement adheres to our policy

The data that support the findings of this study have been anonymized, defaced and converted to the BIDS format 71 using MNE-BIDS 0.8 72. Source data are provided with this paper. The behavioral and MEG data generated in this study and the custom codes used to analyze the data have been deposited in the OpenNeuro database under accession code https://openneuro.org/datasets/ds003922.

Field-specific reporting

Please select the one below that is the best fit for your research. If you are not sure, read the appropriate sections before making your selection.

☒ Life sciences ☐ Behavioural & social sciences ☐ Ecological, evolutionary & environmental sciences

For a reference copy of the document with all sections, see nature.com/documents/nr-reporting-summary-flat.pdf

Life sciences study design

All studies must disclose on these points even when the disclosure is negative.

Sample size	The sample size was determined based on the basis of the orginial paper reporting the Multisensory Correlation Detector (Parise & Ernst, 2016) and is comparable to studies using similar stimuli and paradigm (for example Cao et al., 2019). A sensitivity analysis was conducted and is available in Fig. Supp. 9.
Data exclusions	Epochs contaminated by artificats were rejeted based on peak-to-peak amplitude.
Replication	The behavioral results replicate the ability of the Multisensory Correlation Detector to predict both causality and temporal order judgments of audiovisual sequences as presented in Parise & Ernst (2016).
Randomization	n/a (no group comparison)
Blinding	n/a (no group comparison)

Reporting for specific materials, systems and methods

We require information from authors about some types of materials, experimental systems and methods used in many studies. Here, indicate whether each material, system or method listed is relevant to your study. If you are not sure if a list item applies to your research, read the appropriate section before selecting a response.

Materials & experimental systems

n/a	Involved in the study	
☒	☐	Antibodies
☒	☐	Eukaryotic cell lines
☒	☐	Palaeontology and archaeology
☒	☐	Animals and other organisms
☐	☒	Human research participants
☒	☐	Clinical data
☒	☐	Dual use research of concern

Methods

n/a	Involved in the study	
☒	☐	ChIP-seq
☒	☐	Flow cytometry
☐	☒	MRI-based neuroimaging

Human research participants

Policy information about studies involving human research participants

Population characteristics	Gender: 10 females, 3 males<br>Age: mean 25.8 y., range 22-34 y.<br>All were right-handed, had normal hearing and normal or correted-to-normal vision
Recruitment	Participants were recruited by mailing lists.
Ethics oversight	Local Ethics Committee on Human Research at Neurospin (Gif-sur-Yvette, France)

Note that full information on the approval of the study protocol must also be provided in the manuscript.

Magnetic resonance imaging

Experimental design

Design type	Resting-state
Design specifications	466 s. of resting

Behavioral performance measures: n/a

Acquisition

Imaging type(s): Structural MRI

Field strength: 3 T.

Sequence & imaging parameters: Voxel size: 1.0 x 1.0 x 1.1 mm.
Repetition time: 2300 ms.
Echo time: 2.98 ms.

Area of acquisition: Whole brain

Diffusion MRI: ☐ Used ☒ Not used

Preprocessing

Preprocessing software: FreeSurfer with default parameters.

Normalization: Default parameters.

Normalization template: Default parameters.

Noise and artifact removal: Default parameters.

Volume censoring: Default parameters.

Statistical modeling & inference

Model type and settings: n/a

Effect(s) tested: n/a

Specify type of analysis: ☒ Whole brain ☐ ROI-based ☐ Both

Statistic type for inference (See Eklund et al. 2016): n/a

Correction: n/a

Models & analysis

n/a	Involved in the study
☒	☐ Functional and/or effective connectivity
☒	☐ Graph analysis
☒	☐ Multivariate modeling or predictive analysis
